# Hydrogel Microsphere-Based Alveolar Models for Toxicity Assessment and Pathogen Infection Studies

**DOI:** 10.3390/bioengineering13010017

**Published:** 2025-12-25

**Authors:** Chang Zhou, Jingyuan Ji, Meiling Fu, Yuhui Tang, Yuan Liu, Yang Zheng, Yuan Pang

**Affiliations:** 1 Biomanufacturing Center, Department of Mechanical Engineering, Tsinghua University, Haidian District, Beijing 100084, China; zhou-c23@mails.tsinghua.edu.cn (C.Z.); jijy18@tsinghua.org.cn (J.J.); fumeiling@mail.tsinghua.edu.cn (M.F.); 2 The State Key Laboratory of Membrane Biology, Tsinghua-Peking Center for Life Sciences, School of Life Sciences, Tsinghua University, Haidian District, Beijing 100084, China; tangyuhui58@gmail.com (Y.T.); liu-yuan@tsinghua.edu.cn (Y.L.); 3 Senior Department of Orthopaedics, Fourth Medical Center of PLA General Hospital, No. 51 Fucheng Road, Beijing 100048, China; zhengyang@301hospital.com.cn

**Keywords:** hydrogel microspheres, alveolar model, toxicity assessment, pathogen infection, alternative to animal experiments

## Abstract

The alveolar epithelium plays a critical role in respiratory function, facilitating air exchange and serving as a barrier against inhaled pathogens. Its unique three-dimensional architecture, in which epithelial cells grow on spherical alveolar structures, significantly increases the surface area-to-volume ratio for efficient gas exchange but poses challenges for in vitro reconstruction. Here, we present a biomimetic alveolar model based on gelatin methacryloyl (GelMA) hydrogel microspheres with precisely controlled sizes and composition fabricated via microfluidic technology. These microspheres function as micro-scaffolds for cell adhesion and growth, and an oxygen-permeable honeycomb microwell array facilitates the rapid assembly of cell-laden microspheres into physiologically relevant alveolar-like structures. Using this model, the effects of toxic gas exposure and pathogen infection, and demonstrated its potential use for both basic physiological studies and pathological applications, was investigated. This system recapitulates key features of the alveolar microenvironment and offers a versatile platform for respiratory research and drug screening.

## 1. Introduction

The alveolar epithelium plays a critical role in respiratory function, facilitating air exchange and serving as a barrier against inhaled pathogens. Traditional in vitro alveolar models, which rely primarily on two-dimensional (2D) monolayer cultures or Transwell-based systems, have provided foundational insights into lung physiology and disease [1,2,3]. However, they often fail to recapitulate the three-dimensional (3D) microenvironment, cell–matrix interactions, and mechanical cues present in native alveolar tissue, limiting their physiological relevance.

Recent advances in biomaterials and tissue engineering have enabled the development of more sophisticated 3D alveolar models. Earlier, scientists utilized 3D bioprinting technology to construct alveolar models through the layer-by-layer printing of key alveolar cells. Such models have potential for batch production and can be applied in high-throughput drug evaluation studies [4]. However, limitations of printing precision hinder their ability to accurately replicate the intricate structure of alveoli. With advancements in microfluidic technology, alveolar models constructed within microfluidic chips have emerged. Through ingenious structural designs, porous polydimethylsiloxane (PDMS) membranes can mimic the in vivo air–blood barrier, allowing culture of vascular endothelial cells and alveolar epithelial cells on opposite sides to restore the physiological architecture [1]. Moreover, such chip-based models have enabled studies on SARS-CoV-2, a virus that primarily targets the respiratory system [5], and other common lung diseases [6,7]. As the understanding of how structural cues regulate cellular behavior deepens, it has become clear that surface curvature at scales approaching cellular dimensions can influence cell behavior. This effect is mediated by direct effects on cell adhesion domain proteins, which indirectly modulate related signaling pathways [8,9]. In another study, the incorporation of microelectrodes on a chip enabled the stimulation and signal acquisition of cardiomyocytes, thereby allowing for the collection of mechanical force signals. The application of electrical stimulation significantly enhanced functional performance by modulating the dynamics of tissue organization and contractile development [10]. These findings collectively underscore the critical role of physical factors, such as structure and mechanics, in tissue functionalization. In the field of in vitro alveolar model reconstruction, alveolus-mimicking models incorporating curved structures have gradually gained attention. In one study, thermally pressed polycarbonate films were used to create hemispherical microstructures with surface curvature, serving as micro-scaffolds for alveolar epithelial cell growth. Distinct cellular responses to these curvature features were observed [11]. In another study, hydrogel microspheres were employed as sacrificial templates to generate curved cavity structures within gelatin methacryloyl (GelMA) hydrogels. This allowed cells to adhere to the curved GelMA surfaces, thereby resulting in the generation of alveolar models [12].

Hydrogels are widely utilized for the construction of in vitro three-dimensional tissue models owing to their excellent biocompatibility and tunable formability. Compared with conventional models generated via bioprinting, hydrogel microsphere-based 3D culture systems can better recapitulate the in vivo microenvironment by facilitating essential cell–extracellular matrix interactions. In drug delivery applications, hydrogel microspheres can serve as drug carriers, enabling targeted and localized drug administration [13]. Their small size makes them particularly suitable for injectable delivery formats [14]. Furthermore, cell-laden microcarriers have potential for the high-throughput fabrication of standardized and reproducible delivery systems, while maintaining excellent stability [15,16]. These observations suggest that encapsulating therapeutic cells within hydrogels is a promising delivery strategy. In osteoarthritis research, multipotent stromal cell therapy has garnered significant attention. Preparing hydrogel microspheres loaded with pluripotent stem cells allows for efficient cell encapsulation, stable retention, and enhanced therapeutic efficacy [17].

Hydrogel microspheres loaded with tumor cells are commonly used to model tumors. The three-dimensional spheroid environment facilitates studies on the effects of mechanical signals on tumor cell growth and progression, the emergence of necrotic cores due to hypoxia [18], and tumor stem cell stemness and related signaling pathways [19]. Furthermore, encapsulating cells within hydrogel microspheres enables the construction of cell-laden micro-units. This approach aligns with the bottom-up tissue engineering strategy, allowing researchers to build basic tissue units with defined spatial structures and functions, thereby providing a foundation for the construction and application of more complex three-dimensional models [20].

**Figure 1:**
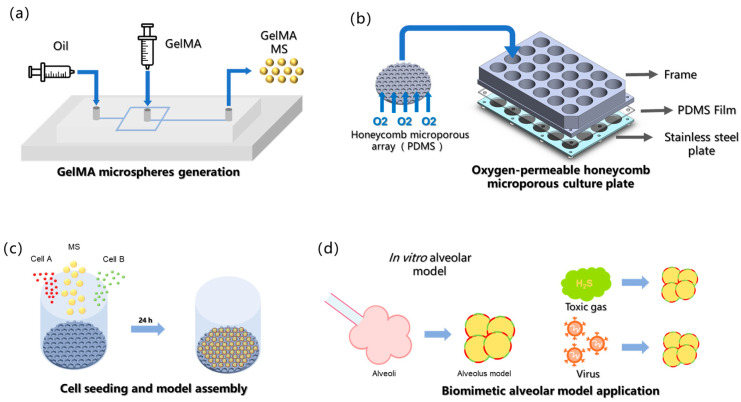
Conceptual framework of current study. (**a**) GelMA microspheres generation. (**b**) Oxygen-permeable honeycomb microporous culture plate. (**c**) Cell seeding (both cell type A and cell type B can be co-seeded with GelMA MS). (**d**) The biomimetic alveolar model is employed for studying gaseous toxin and pathogenic infection in vitro.

The fabrication of uniformly sized hydrogel microspheres on a hundred-micrometer scale using microfluidic chips has emerged as a mature and scalable technology [6,21,22]. In this study, gelatin methacryloyl (GelMA) hydrogel was selected as the biomaterial for microsphere carrier fabrication using a microfluidic chip [23] (Figure 1a) because of its excellent cell compatibility, adhesion capacity, and mechanical properties, which support cell adhesion to its surface and provide a structural scaffold for cell growth [24]. Notably, this platform enables the incorporation of multiple cell types in a spatially controlled manner to investigate intercellular communication, a critical feature unattainable in traditional culture systems.

In this study, a novel approach was developed for constructing an alveoli-mimetic model using hydrogel microspheres as cell-loaded microcarriers. Compared with conventional encapsulation methods, wherein cells are embedded within hydrogel matrices [23,25], our strategy involves attaching cells to the exterior surface of microspheres to form a curved monolayer cellular membrane structure that more accurately recapitulates the native growth pattern of alveolar epithelial cells, which naturally organize as curved monolayers in vivo. It is posited that such 3D architectural fidelity is crucial for cells to maintain physiological functions comparable to their in vivo counterparts, ultimately leading to the superior reconstruction of alveolar models under in vitro conditions [8]. The surface loading paradigm represents a significant advancement over traditional encapsulation techniques in that it better preserves the characteristic curvature and mechanical microenvironment, which are essential for proper alveolar epithelial cell function. Since our surface loading strategy requires post-fabrication cell attachment after microsphere cross-linking, a honeycomb-inspired micropore array fabricated by replica molding of polydimethylsiloxane (PDMS) onto photolithographically patterned silicon wafers was used. The PDMS substrate was used owing to its exceptional oxygen permeability, which ensures an adequate oxygen supply during cell-microsphere assembly (Figure 1b) [26]. Microporous arrays with a diameter of 326 μm were used to provide sufficient space for the assembly of cells and microsphere carriers; moreover, the microporous array surfaces were hydrophobically treated to discourage cell attachment, thereby directing cells to aggregate around the hydrogel microspheres and achieve specific cell adhesion on the microsphere surfaces (Figure 1c). Finally, this model was applied to in vitro inhalation gas toxicity assessment and pathogen infection studies (Figure 1d).

## 2. Materials and Methods

### 2.1. GelMA Microsphere Preparation

GelMA (EFL-GM-60; Suzhou Yongqinquan Intelligent Equipment Co., Ltd., Suzhou, China) was dissolved in a photoinitiator solution of lithium phenyl-2,4,6-trimethylbenzoylphosphinate at a concentration of 0.25% (*w*/*v*) and prepared as a sterile 5% (*w*/*v*) solution filtered through 0.22 μm membranes. Microfluidic synthesis was performed using mineral oil containing 2% Span 80 as the continuous phase (2.5 mL/h flow rate) to shear the aqueous GelMA phase (0.25 mL/h) into hundred-micrometer scale microspheres. Immediate photopolymerization was achieved with 405 nm UV irradiation (2500 mW/cm^2^ intensity) at the collection outlet. GYY4137 (Cat. No. SML0100; Sigma-Aldrich, Shanghai, China) (0.5 g) was dissolved in 10% GelMA solution, vortexed thoroughly, and filtered (0.22 μm) prior to microfluidic generation of GYY-loaded microspheres.

### 2.2. Honeycomb Array Preparation

All components, including the PDMS frame, stainless-steel plate, and microporous array, were autoclave sterilized. The honeycomb array was treated with oxygen plasma for 20 s and coated with a 3:2 (*v*/*v*) artificial cell membrane/ethanol solution to generate a hydrophobic surface. The assembled platform is compatible with commercial 24-well plates and could be used with sterile lids.

### 2.3. Cell Culture

A549 human adenocarcinoma cells (CCL-185; American Type Culture Collection [ATCC], Manassas, VA, USA) were cultured in Dulbecco’s modified Eagle’s medium (DMEM; Gibco, Waltham, MA, USA) containing 10% fetal bovine serum (FBS; Hyclone, Logan, UT, USA), 1% penicillin/streptomycin (100 U/mL; BI), 1% GlutaMAX (Gibco), and 1% non-essential amino acids (Gibco). HUVECs (ATCC) were cultured in complete DMEM containing growth factors. 293-ACE2 overexpressing cells, SARS-CoV-2 (D614G) GFP Luc pseudovirus, and anti-2019-nCoVS-hIgG1 neutralizing antibody (all from Novoprotein, Shanghai, China) were cultured in DMEM with 10% FBS and 50 μg/mL G418 (Geneticin).

The *mouse* lung tissues were isolated and placed in DBPase solution for digestion at room temperature for 45 min. Subsequently, the tissues were transferred to 5 mL of digestion solution containing DNase I (prepared in HEPES-buffered DMEM), thoroughly minced with surgical scissors, and vigorously pipetted. The mixture was then incubated at room temperature for an additional 10 min to facilitate digestion. The resulting cell suspension was sequentially filtered through 100 μm, 70 μm, and 40 μm cell strainers. The filtrate was collected and centrifuged at 200× *g* for 10 min at 4 °C to pellet the cells. To remove red blood cells, the cell pellet was resuspended in ACK lysing buffer and incubated at room temperature for 5 min. After lysis, an excess volume of PBS containing Primulan was added to wash the cells, followed by another centrifugation step under the same conditions (200× *g*, 10 min, 4 °C). This washing procedure was repeated twice to ensure the complete removal of impurities. The final purified pellet of primary lung cells was obtained for subsequent experiments.

### 2.4. Assembly of Cells and GelMA Microspheres

GelMA microspheres were resuspended in culture medium (5000 microspheres/mL) and combined with cell suspensions (5 × 10^5^ cells/mL). After adding 1 mL of the microsphere suspension to the array and allowing 5 min of sedimentation, 1 mL of the cell suspension was gently added prior to incubation under standard culture conditions. For the co-seeding of the two cell types, a uniform mixture of the cells was added to the honeycomb microwells containing pre-sedimented GelMA microspheres (MS), enabling the co-adhesion of multiple cell types to the MS surface.

### 2.5. H_2_S Release Quantification

The methylene blue method was employed, wherein zinc acetate (5%) trapped H_2_S as ZnS, which reacted with N,N-dimethyl-p-phenylenediamine (0.2%) and ferric ammonium sulfate (10%) to generate a chromophore measurable at 670 nm. NaHS (20 mM) was used as the calibration standard.

### 2.6. Gene Expression Profiling

After RNA extraction and reverse transcription, A549 cells exposed to H_2_S for 24 h in the alveolar model were analyzed using qPCR for surfactant protein genes (SFTPA1, SFTPB, SFTPC, and SFTPD) and apoptosis markers (BAX and BCL2).

### 2.7. Pseudovirus Infection and Anti-Body Neutralization

Endothelial spheroids created via a 1:1 co-culture of ACE2-overexpressing 293 cells and HUVECs on microspheres were infected with SARS-CoV-2 (D614G) GFP Luc pseudovirus after 48 h of maturation. The antibody concentrations (100, 25, 10, 5, and 0 μg/mL) used for neutralization assays were based on 2D-effective doses, and infection was monitored for 48 h.

## 3. Results and Discussion

**Figure 2:**
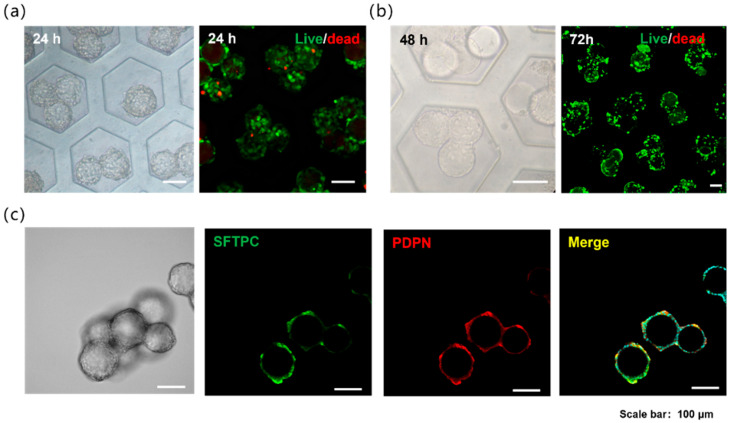
(**a**) Alveolar models of GelMA microspheres loaded with A549 cells in the microwells and the cell live/dead staining. (**b**) Alveolar models of GelMA microspheres loaded with primary *mouse* alveolar epithelial cells in the microwells and the cell live/dead staining. (**c**) Immunofluorescence staining of the alveolar models with primary *mouse* alveolar epithelial cells, red is PDPN (Podoplanin, AT1 cell marker), green is SFTPC (Surfactant Protein C, AT2 cell marker).

The microporous surface was subjected to hydrophobic treatment; thus, the cells settling into the micropores did not adhere to the walls but instead aggregated toward the GelMA microspheres, ultimately adhering to their surface. This process was completed within 24 h. A549 cells are commonly used in the construction of in vitro alveolar models, primarily because of their ability to express alveolar surfactants. Pulmonary surfactant (PS), a phospholipid-protein complex synthesized and secreted by type II alveolar epithelial cells, consists of 70–80% phospholipids, 10% proteins, and 10% neutral lipids. Similarly to type II alveolar epithelial cells, A549 cells retain their proliferative capacity, both of which are key functional characteristics of type II pneumocytes. Following the assembly technique described above, the A549 cells uniformly adhered to the microsphere surface while maintaining high viability (Figure 2a). Primary *mouse* alveolar epithelial cells isolated via enzymatic digestion were seeded and cultured using the same method, and these cells also adhered uniformly to the microspheres and exhibited high viability (Figure 2b). In these results, the cell-adherent hydrogel microspheres remained solid and non-hollow. Because the digested *mouse* alveolar cell population contained multiple cell types, including alveolar type I and type II cells, immunofluorescence staining was performed to label specific markers. The results confirmed the presence of both type I and II alveolar epithelial cells growing on the microspheres (Figure 2c). Given that these two cells’ types constitute the major alveolar epithelial population, this distribution closely mimics physiological conditions.

**Figure 3:**
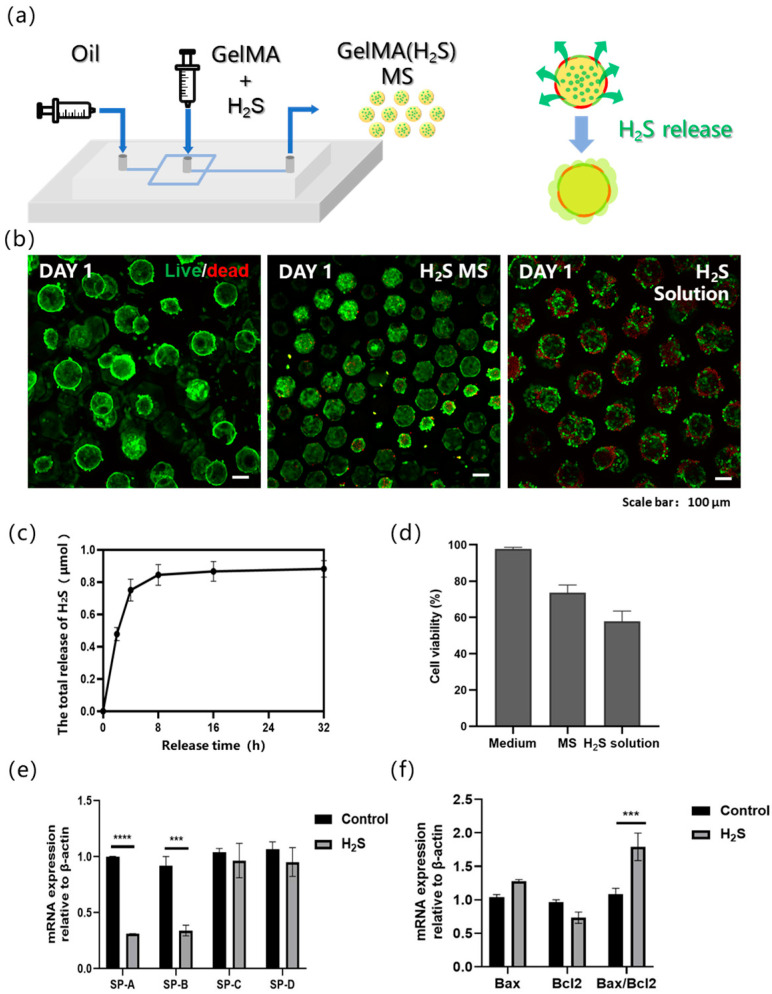
(**a**) Schematic illustration of hydrogen sulfide (H_2_S)-loaded microsphere fabrication and H_2_S release process. The yellow spherical parts are schematic representations of the prepared hydrogel microspheres. The green components encapsulated within the yellow spheres are H_2_S carriers. The red-green intercalated structures surrounding the yellow spheres serve as schematic representations of cells, and the arrows indicate the directional release of H_2_S from the interior to the exterior. (**b**) Live/dead staining of A549 cells on alveolar models under different H_2_S treatment conditions, from left to right: non-H_2_S group, H_2_S-releasing microsphere group, and H_2_S solution group. (**c**) The variation in H_2_S release over time in GYY-loaded microspheres. (**d**) Statistical analysis of cell viability in the control group, microsphere-release group, and solution-release group. (**e**) Influence of H_2_S on gene expression of pulmonary surfactant in three-dimensional alveolar model. (**f**) Influence of H_2_S on gene expression of apoptosis in three-dimensional alveolar model. n = 3, independent experiments, *** *p* < 0.001; **** *p* < 0.0001.

The alveoli serve as a critical physiological barrier in the human body; however, they are highly susceptible to damage from environmental substances and microorganisms during respiration, leading to acute injury, chronic disorders, and systemic inflammatory diseases [27,28,29]. To investigate the cellular damage in alveoli caused by exposure to toxic gases, the outward diffusion of inhaled toxicants and their subsequent effect on alveolar cells was simulated (Figure 3a). To replicate the diffusion process of toxic gases within the alveoli, GYY4137 was incorporated (an H_2_S donor) into the GelMA hydrogel microspheres during fabrication. GYY4137 is water-soluble and enables sustained H_2_S release through hydrolysis. The H_2_S release kinetics of the prepared GYY-loaded microspheres was quantified and found that they released approximately 0.8 μmol of H_2_S over 24 h (Figure 3c). Accordingly, the cell viability in the model was assessed after 24 h of co-culture with the microspheres, in addition to hydrogel microspheres, the images also contain cell-derived microspheres formed by spontaneous cell aggregation; moreover, the apparent size variations in the microspheres are caused by differences in the quantity and adhesion state of cells on the microspheres (Figure 3b). Three experimental groups were established: (1) a control group with culture medium only, (2) a microsphere-release group containing H_2_S-releasing microspheres, and (3) a solution-release group in which GYY was directly added to the medium. The control group exhibited negligible cell death. Compared with the microsphere-release group, which exhibited considerable cell death, the solution-release group exhibited a significantly higher mortality rate, accompanied by pronounced cell detachment and increased contour diameter of the cell-laden microspheres. Importantly, the microspheres maintained their structural integrity, and the released H_2_S induced localized cell death in nearby cell spheroids, which was not observed in the control group (Figure 3b). Furthermore, statistical analysis of cell viability based on fluorescence area quantification revealed that the cell damage induced by H_2_S released from hydrogel microspheres was less severe, with significantly higher cell viability, compared to the direct exposure of cells to H_2_S added in solution (Figure 3d). These findings confirm that the microsphere-based release model successfully mimics the inward-to-outward diffusion of toxic gases and their subsequent cytotoxic effects.

Real-time quantitative PCR (qPCR), a widely used method for quantifying target gene expression, was employed to investigate H_2_S-induced alterations in gene expression in alveolar type II epithelial cells. Specifically, the expression levels of surfactant-associated genes in A549 cells were analyzed 24 h after H_2_S exposure in the bionic alveolar model. Pulmonary surfactant protein is critical for maintaining alveolar surface tension and preventing alveolar collapse. It exhibits high specificity and expression levels in lung epithelial cells. The pulmonary surfactant proteins include surfactant protein A (SP-A), SP-B, SP-C, and SP-D, of which SP-A is the most abundant, accounting for 50–70% of the total surfactant proteins, and SP-B is the most functionally critical but constitutes only approximately 10%. SP-B and SP-C are small hydrophobic proteins that are primarily involved in regulating surface tension, whereas SP-A and SP-D are large hydrophilic proteins that contribute to pulmonary host defense. Compared with the micro-lung chip model, our model also demonstrated high expression of the SP protein family in A549 cells [30]. Our results revealed significant downregulation of SP-A and SP-B (Figure 3e) and upregulation of apoptosis-related genes (Figure 3f) in A549 cells after a 24 h H_2_S exposure, indicating that H_2_S impairs the functional capacity of type II alveolar epithelial cells. This suppression of surfactant protein expression suggests a potential mechanism by which H_2_S disrupts pulmonary surfactant homeostasis and compromises alveolar function. This injury-induced downregulation was similarly observed in lung chip models that were induced to develop pulmonary fibrosis [30,31].

**Figure 4:**
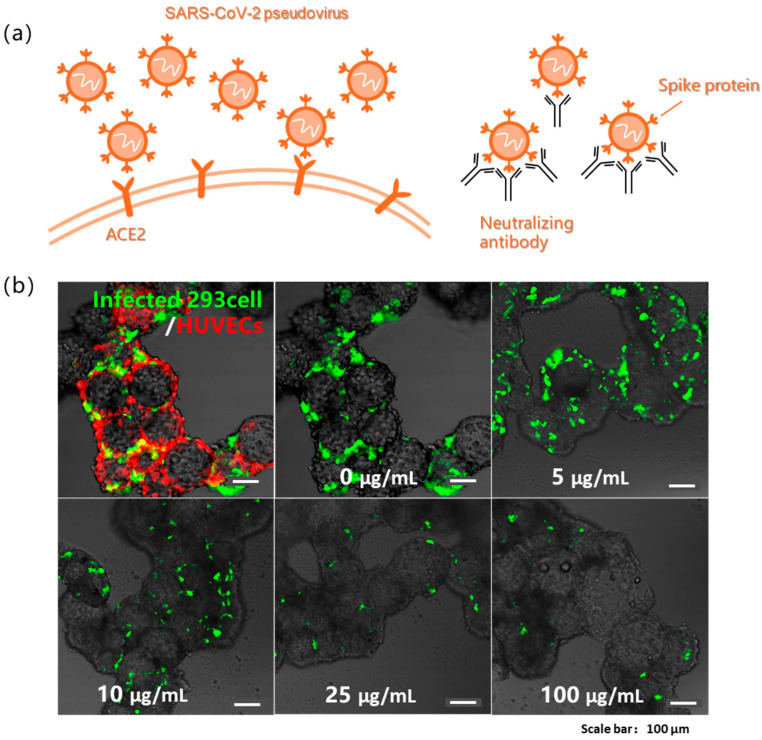
(**a**) Schematic illustration of SARS-CoV-2 pseudovirus infection and antibody neutralization. (**b**) Pseudovirus infection in 293 cells and HUVECs cocultured model visualized by GFP+ infected 293 cells (green) and celltracker-labeled HUVECs (red), imaged at 48 h post-infection, and gradient neutralization assay quantifying pseudovirus infection efficiency in 293 cells at serially diluted antibody concentrations.

The severe acute respiratory syndrome coronavirus 2 (SARS-CoV-2) pseudovirus was also used for in vitro infection studies (Figure 4a) [5,32]. In this study, endothelialized mic spheroids were constructed by co-culturing ACE2-overexpressing 293 cells with vascular endothelial cells for 48 h, followed by infection with the SARS-CoV-2 pseudovirus for another 48 h. Vascular endothelial cells were incorporated based on established research demonstrating that SARS-CoV-2 can induce microvascular injury, thereby compromising blood–brain barrier integrity and leading to neural damage [33]. Fluorescence images revealed that 293 cells exhibited distinct green fluorescence, indicating successful infection with the pseudovirus. HUVECs (human umbilical vein endothelial cells) on the microspheres were pre-labeled with a red live cell tracker (CellTracker) for visualization. Both cell types adhered well to the surface of the microspheres. The cystic spheroid structure remained observable throughout the culture period, demonstrating the potential of this model for in vitro 3D viral infection studies.

In virology research and drug development, neutralizing antibodies are commonly investigated as antiviral agents to study their inhibitory effects on viral infections or to develop novel virus-specific neutralizing antibodies [34,35]. The pseudovirus neutralization assay used in this study was based on this principle. Endothelial multicellular cystic spheroids were cultured for 48 h in microplates and subjected to neutralization tests. Five different antibody concentrations (100, 25, 10, 5, and 0 μg/mL) were prepared for the 3D experiment. In 2D cell models, the half inhibitory concentration (IC50) of neutralizing antibodies required to block pseudovirus infection was 25 ng/mL. However, testing based on our three-dimensional model revealed a significant increase in the required concentration, with the IC50 for half-maximal infection inhibition, determined by curve fitting, to be 5.86 μg/mL (Figure 4b). These results suggest that substantially higher neutralizing antibody concentrations are required to effectively inhibit pseudovirus infection in the 3D model, highlighting the critical differences in drug susceptibility between the dimensional culture systems.

## 4. Conclusions

In this study, a biomimetic alveolar model was developed using GelMA hydrogel microspheres and a honeycomb array to recapitulate the key structural and functional features of the alveolar epithelium. The model was successfully applied to simulate toxic gas exposure, through which localized cytotoxicity and impaired surfactant protein expression were demonstrated. It was also utilized in pathogen infection studies, where a significantly higher antibody neutralization threshold in 3D than in 2D cultures was revealed. This 3D biomimetic alveolar model holds promising future applications, particularly in respiratory disease research and drug development. With further technological refinement, this model may facilitate high-throughput automated analysis, accelerating advancements in respiratory medicine research.

## Data Availability

The original contributions presented in this study are included in the article. Further inquiries can be directed to the corresponding author.
